# Dataset for the estimation of costs for direct contact condenser

**DOI:** 10.1016/j.dib.2018.08.042

**Published:** 2018-08-22

**Authors:** Dunyu Liu, Jing Jin, Ming Gao, Zhibo Xiong, Rohan Stanger, Terry Wall

**Affiliations:** a School of Energy and Power Engineering, University of Shanghai for Science and Technology, Shanghai 200093, China; b Chemical Engineering, University of Newcastle, 2308, Australia

**Keywords:** Direct contact condenser, Packed bed, Capital cost, Annual cost

## Abstract

The dataset on equations and procedures for the estimation of detailed capital and annual costs for direct contact condenser are presented. Full dataset on four design cases relevant to the comparisons on the costs of air and oxy-fuel direct contact condenser is given. The data are presented in this format to allow the comparison with those from other researchers in this field. The data presented are related to the article entitled “A comparative study on the design of direct contact condenser for air and oxy-fuel combustion flue gas based on Callide Oxy-fuel Project” (Liu et al., 2018) [1].

## Nomenclature

*C*_e_: cost of electricity
*Cs*: cost of solvent
*C*c: cost of chemical
*C*_OL_: cost of operation labor
*C*_Sup_: cost of supervisor
*C*_ML_: cost of maintenance labor
*C*_M_: cost of material
$C_{n}$: projected cost in the year n
$C_{m}$: known cost in the year m
*C*_ww_: solvent disposal costs
*D*: column diameter, ft
*Energy*_fan_: energy required for the fan
*Energy*_pump_: energy required for the pump
*G*: gas mass velocity, lb/(s ft^2^)
*G*_1_: molar rate of flue gas
$G_{i}$: gas flow rate
*H*_tower_: tower height, ft
*H*_pack_: packing height, ft
$I_{n}$: index in the year n
$I_{m}$: index in the year m
L: water mass flux, kg/m^2^ s
*L*_1_: liquid mass velocity lb/(s ft^2^)
$L_{i}$: water flow rate, gpm
*P*_SO2_: inlet partial pressure of SO_2_
P: total pressure drop through the system
*Pressure*: working pump pressure
*S*: tower surface area, ft^2^
TTC: total cost of the tower

**Greek letters**
α: packing parameter
β: packing parameter
$\rho_{G}$: gas density, lb/ft^3^
ΔP: pressure drop, in H_2_O/ft packing
$\eta_{SO_{2}}$: SO_2_ removal efficiency
ε: fan motor efficiency

**Abbreviations**
AC: air combustion;
OC: oxy-fuel combustion;
*TCI*: total capital investment;
*PEC*: purchased equipment cost;
*DIC*: direct installation costs;
*IC*: indirect installation cost;
CF: cost factor;
*PC*: packing cost;
*UPC*: unit packing cost;
*OLC*: operating labor costs;
*MLC*: maintenance labor cost;
*AEC*: auxiliary equipment costs;
*DIC*: direct installation costs;
*DC*: direct annual cost;
*WF*: waste fraction;
*SUC*: solvent unit cost;
*AOH*: annual operating hours;
*TTC*: total tower cost;
*PV*: packing cost;
*UPC*: unit packing cost;
*CUC*: chemical unit cost;
*AOH*: annual operating hours;
*CUPH*: chemical used per hour;
*MRC*: molar rate of caustic;
*MFR*_Na2SO4_: mass flow rate of Na_2_SO_4_;
*M*_Na2SO4_: molecular weight of Na_2_SO_4_;
*WVFR*: wastewater volume flow rate;
*DSS*: density of Na_2_SO_4_ solution;
*SDC*: unit solvent disposal costs;
*COE*: unit cost of electricity;
*CRC*: capital recovery cost;
*TAC*: total annual cost;

**Specifications Table**

**Table:** 

**Subject area**	*Chemical engineering, Environmental engineering, Environmental science*
**More specific subject area**	*Direct contact condenser, Oxy-fuel combustion capture, Power plant*
**Type of data**	*Tables, Equations*
**How data was acquired**	*The diameter and packing height of direct contact condenser were obtained by programing based on MATLAB. Cost data was obtained based on empirical formulas and realized in Excel software.*
**Data format**	*Raw and analyzed data*
**Experimental factors**	*Two flue gas flue rates, two flue gas compositions, four L/G ratios were studied and optimized factors included normalized capital and annual costs.*
**Experimental features**	*The impacts of L/G ratio on capital and annual costs for both oxy-fuel and air-fuel combustion were identified.*
**Data source location**	*Shanghai, China*
**Data accessibility**	*The data are available with this article*
**Related research article**	Liu et al. [1]

**Value of the data**

• The data on the calculation methodology of capital and annual costs for direct contact condenser can potentially be used by researchers interested in the cost analysis of power plant.
• Breakdown costs of direct contact condenser are useful for researchers to compare similar size of units.
• Cost data is useful for these researches who have interest in the reduction of unit cost based on the proportions of breakdown costs with respect to total unit cost.

### 1 Data

The data in this article provides the detailed calculation formulas necessary to obtain capital and annual costs for DCC. Selection of these formulas are important for cost analysis and in turn relevant for the design of DCC. Four design cases in terms of different L/G ratios are given with two cases for both air and oxy-fuel combustion flue gases. The required parameters for the cost analysis include packing parameters, condenser height and diameter. With these, detailed breakdown costs data are then provided for four cases.

The whole Section 2 describes five steps involved for the calculation of costs. Based on the detailed formulas and parameters, Table1 provides the data on the breakdown costs for capital and annual costs for four cases with different L/G ratios.

**Table 1:** Calculation of cost parameters.

Parameters	Units	Equations	Case 1	Case 2	Case 3	Case 4
		**Total Capital Investments**				
*L*/*G*	kmol/kmol	*L*/*G*(dry molar flow rate)	5.2	2.5	6.7	4.3
*H*_pack1_	m	Simulation	0.296	0.417	0.326	0.461
ft to m factor	m/ft	1 ft = 0.3048 m	0.3048	0.3048	0.3048	0.3048
*H*_pack2_	ft	*H*_pack1_ × 0.3048	0.971	1.368	1.070	1.512
*D*_1_	m	Simulation	8.65	8.08	3.86	3.67
*D*_2_	ft	*D*_1_ × 0.3048	28.379	26.509	12.664	12.041
*S*_cross-section_	ft^2^	π × (*D*_2_)^2^*/*4	632.527	551.912	125.957	113.862
*V*_pack_	ft^3^	π × (*D*_2_)^2^*H*_pack2_*/*4	614.265	755.077	134.718	172.213
*G*	kmol/s	Design (dry basis)	1.675	1.675	0.297	0.297
*G*_0_	kmol/s	Design (wet basis)	1.821	1.821	0.379	0.379
*L*	kmol/s	*G*×( *L*/*G*)	8.71	4.188	1.990	1.277
*H*_tower_	ft	1.40 *H*_pack_ + 1.02 *D*_*2*_ +2.81	33.116	31.765	17.225	17.209
*S*	ft^2^	π *D*_2_ × ( *H*_tower_+ *D*_2_/2)	4217.499	3749.146	937.183	878.667
*CF*	–	Cost factor selected	1.5	1.5	1.5	1.5
*TTC*	$	115*CF* × *S*	727,518	646,728	161,664	151,570
*UPC*	$/ft^3^	Selected	40	40	40	40
*PC*	$	*UPC* × *V*_pack_	24,570	30,203	5389	6889
*AEC*	$	0.15*EC*	132,721	119,458	29,480	27,963
*EC*	$	*TTC*+*PC* +*AEC*	884,810	796,389	196,533	186,422
*PEC*	$	1.18*EC*	1044,076	939,739	231,908	219,978
*DIC*	$	0.85*PEC*	887,465	798,778	197,122	186,981
*IC*	$	0.35*PEC*	365,426	328,909	81,168	76,992
*TCI*	$	2.2*PEC*	2296,969	2067,426	510,199	483.951
*I*_m_	–	Index in the year m	361.3	361.3	361.3	361.3
*I*_n_	–	Index in the year n	588.4	588.4	588.4	588.4
*TCI*_new_	$	*TCI×I*_m_/ *I*_n_	3740,759	3366,934	830,891	788,145
		**Direct Annual Costs**				
*P*_CO2_	–	Design	0.138	0.138	0.566	0.566
*P*_N2_	–	Design	0.741	0.741	0.176	0.176
*P*_H2O_	–	Design	0.08	0.08	0.216	0.216
*P*_O2_	–	Design	0.041	0.041	0.042	0.042
*P*_SO2_	–	Design	220×10^-6^	220×10^-6^	890×10^-6^	890×10^-6^
*M*_CO2_	g/mol	Molecular weight	44	44	44	44
*M*_N2_	g/mol	Molecular weight	28	28	28	28
*M*_H2O_	g/mol	Molecular weight	18	18	18	18
*M*_O2_	g/mol	Molecular weight	32	32	32	32
*M*	g/mol	*M*_CO2_*P*_CO2_+*M*_N2_*P*_N2_+*M*_H2O_*P*_H2O_+*M*_O2_*P*_O2_	29.57	29.57	35.064	35.064
ρ_N2_	kg/m^3^	1.25 × 273/(273+145)	0.816	0.816	0.816	0.816
ρ_CO2_	kg/m^3^	1.96 × 273/(273+145)	1.280	1.280	1.280	1.280
ρ_O2_	kg/m^3^	1.43 × 273/(273+145)	0.934	0.934	0.934	0.934
ρ_H2O_	kg/m^3^	ρ_N2_ ×1 8/28	0.525	0.525	0.525	0.525
ρ_G_	kg/m^3^	*M*_CO2_ ρ_CO2_ + *M*_N2_ ρ_N2_ + *M*_H2O_ ρ_H2O_ + *M*_O2_ ρ_O2_	0.862	0.862	1.021	1.021
ρ_G1_	lb/ft^3^	ρ_G_/16	0.054	0.054	0.064	0.064
lb to g conversion ratio	g/lb	1 lb = 453.59 g	453.59	453.59	453.59	453.59
*G*_1_	lb/s	*G*_0_×1000×*M*/453.59	118.721	118.721	29.298	29.298
*G*_2_	lb/ft^2^/s	*G*_1_/ *S*_cross-section_	0.188	0.215	0.233	0.257
*G*_3_	ft^3^/min	*G*_1_/ ρ_G1_×60	132237.5	132237.5	27552.8	27552.8
*L*_1_	lb/s	*L*×1000×18/453.59	345.642	166.174	78.966	50.680
*L*_2_	lb/ft^2^/s	*L*_1_/ *S*_cross-section_	0.546	0.301	0.627	0.445
*ρ*_L_	lb/ft^3^	992.74(kg/m^3^)/16	62.046	62.046	62.046	62.046
ΔP	inch H_2_O/ft packing	$\alpha 10^{\beta L_{2}}\left(\frac{G_{2}^{2}}{\rho_{G1}}\right)$	0.457	0.530	0.617	0.689
*P*	inch H_2_O	ΔP× *H*_pack2_	0.444	0.725	0.660	1.042
Gallon to liters	L/gallon	1 gallon≈3.785 l				
Gallon to ft^3^	ft^3^/gallon	1 gallon≈0.134 ft^3^				
Gallon to lb	lb/gallon	1 gallon≈0.134 ft^3^× *ρ*_L_	8.294	8.294	8.294	8.294
*L*_3_	gpm	*L*_1_×60/8.294	2500.325	1202.079	571.228	366.609
*AOH*	h	Annual operating hours	8000	8000	8000	8000
*C*_OL_	$	0.5/8 × 8000 × $28.84	14,420	14,420	14,420	14,420
*C*_Sup_	$	15% *C*_OL_	2163	2163	2163	2163
*C*_ML_	$	0.5/8×8000×$24.48	12,240	12,240	12,240	12,240
*C*_M_	$	*C*_ML_	12,240	12,240	12,240	12,240
*MRC*	kmol/s	*0.9G*_0_ × 2 × *P*_SO2_	0.000663	0.000663	0.000476	0.000476
*MRC*_1_	t/hr	*MRC* × 3600 × 40/1000	0.095515	0.095515	0.06851	0.06851
*MRC*_2_	lb/hr	*MRC* × 1000 × 40 × 3600/453.59	210.576	210.576	151.049	151.049
*C*c	$	*MRC*_1_ × 500 × 8000	382,060	382,060	274,057	274,057
*MFR*_Na2SO4_	g/s	*MRC* × 1000 × *M*_Na2SO4_	94.189	94.189	67.563	67.563
*MFR*_Na2SO41_	lb/hr	*MFR*_Na2SO4_/453.59×1000	747.545	747.545	536.224	536.224
*DSS*	g/L	Density of Na_2_SO_4_ solution at 20 °C	1090.5	1090.5	1090.5	1090.5
*DSS*1	lb/gal	*DSS/*453.59×3.785	9.101	9.101	9.101	9.101
*WVFR*	gpm	*MFR*_Na2SO41_/0.1/ *DSS*1/60	13.690	13.690	9.820	9.820
*C*_ww_	$	*WVFR*×60×8000×2/100	131,427	131,427	94,274	94,274
*C*_s_	$	*WVFR*×60×8000×0.6/1000	3943	3943	2828	2828
*Energy*_fan_	kw	$\;\frac{1.17\times 10^{-4}G_{3}P}{0.7}$	9.813	16.035	3.041	4.800
*Energy*_pump_	kw	$\frac{(0.746)\times \left(2.52\times 10^{-4}\right)L_{3}\times 60}{0.7}$	28.202	13.559	6.443	4.135
*Total Energy*	kw	*Energy*_fan+_*Energy*_pump_	38.015	29.594	9.485	8.935
*C*e	$	*Total Energy*×8000×0.675	205,281	159,807	51,216	48,249
*DC*	$	*C*_OL_+*C*_Sup_+*C*_ML_*+C*_M_+*C*c+ C_ww_+*C*_s_+*C*e	763,775	718,300	463,439	460,472
		**Indirect Annual Costs**				
Overhead	$	0.6× (*C*_OL_+ *C*_Sup_+ *C*_ML_+ *C*_M_)	24,638	24,638	24,638	24,638
Administrative charges	$	0.02 *TCI*_new_	74,815	67,339	16,618	15,763
Property tax	$	0.01 *TCI*_new_	37,408	33,669	8309	7881
Insurance	$	0.01 *TCI*_new_	37,408	33,669	8309	7881
Capital recovery	$	0.1315 *TCI*_new_	491,910	442,752	109,262	103,641
*IC*	$	Overhead+Administrative charges+Property tax+Insurance+Capital recovery	666,178	602,067	167,136	159,804
Total annual cost	$	*DC+IC*	1,429,952	1,320,367	630,575	620,277

### 2 Experimental design, materials and methods

#### 2.1 Empirical expressions for equipment

Estimating procedure for total cost involves five steps: (1) determination of facility parameters; (2) design of control system; (3) determination of control system size; (4) estimation of individual components; and (5) estimation of costs for the entire system [2].

The parameters including condenser packing height, condenser diameter, packing characteristics and L/G ratio for estimating the total capital costs for DCC were obtained by simulation [1].

Eq. (1) is used to estimate the total tower height [3].

(1) $$H_{tower}=1.40\;H_{pack}+1.02\;D+2.81$$

Here, *D* is the diameter of condenser, ft; *H*_tower_ tower height, ft; *H*_pack_ packing height, ft.

The tower surface area is expressed by Eq. (2) and this equation assumes the ends of tower are flat and circular.

(2) $$S=\pi D\left(H_{tower}+D/2\right)$$

The pressure drop is estimated by Eq. (3) [4].

(3) $$\Delta P=\alpha 10^{\beta L_{1}}\left(\frac{G^{2}}{\rho_{G}}\right)$$

For Raschig rings Ceramic and a nominal size of 1 in, the value for α and β are 0.53 and 0.22 respectively for packed tower operating below flooding region. *G* is gas mass velocity (respective to tower cross section area), lb/(sec·ft^2^); *L*_1_ is liquid mass velocity lb/(sec·ft^2^); $\rho_{G}$ is gas density, lb/ft^3^. ΔP is the pressure drop, in H_2_O/ft packing.

#### 2.2 Estimation of total capital investment

Total capital investment *TCI* [3] includes purchased equipment cost, *PEC*, direct installation costs, *DIC*, and indirect installation cost, *IC*.

1) Purchased equipment costs (*PEC*)
  The equipment cost is estimated based on the surface area of tower. Total tower cost is estimated by Eq. (4).
  (4) TTC=115CF×S
  Here, TTC is the total cost of the tower using other materials than fiberglass reinforced plastic (*FTP*); *S* is the tower surface area; CF is the cost factor to convert the cost of an FRP tower to a tower constructed by other materials. Commonly used material is 304 Stainless steel, and the cost factor, *CF* is in the range of 1.10–1.75; CF is chosen as 1.5.
  Auxiliary costs incorporate the cost not included in the tower unit. For the packing type of Rasching rings and construction material of ceramic with 1 in. nominal diameter, the packing cost for small volume is chosen as $40/ft^3^.
  The auxiliary equipment costs (*AEC*) not only includes costs for pump, fan and motor, but also includes these for ductwork, piping and others. a factor of 0.15 with respective of EC is used [3], [5]. *EC* is calculated by Eq. (5).
  (5) EC=TTC+PC+AEC
  The calculation of Chemical Engineering Plant Index (CEPI) [6], [7] includes four sub-indexes incorporating equipment, construction labor, building and engineering and supervision and these aspects can be treated as the costs for building the plant. Eq. (6) [8] is used to correlate the equipment costs in 1991 to these in 2017 [6], [8]. Here, CEPI is used escalate *EC*.
  (6) $$C_{n}=\left(I_{n}/I_{m}\right)\times C_{m}$$
  Here, $C_{n}$ is the projected cost in the year n; $C_{m}$ is the known cost in the year m; $I_{n}$ is the index in the year n; and $I_{m}$ is the index in the year m.
  The purchased equipment cost (*PEC*) includes the cost of the tower with packing and auxiliaries (*EC*), instrumentation (0.1*EC*), sales tax (0.03*EC*), and freight (0.05*EC*). Therefore, *PEC* is calculated as follows by Eq. (7) [9].
  (7) PEC=(1+0.1+0.03+0.05)EC=1.18EC
2) Direct installation costs (*DIC*)
  Direct installation costs (*DIC*) include foundations & supports, handling & erection, electrical, piping, insulation and painting. The direct installation costs are estimated as 0.85PEC.
3) Indirect installation costs (*IC*)
  Indirect costs (*IC*) include engineering, construction and field expenses, contractor fees, start-up, performance test, and contingencies. The indirect costs are estimated as 0.35*PEC*.
4) Total capital investment (*TCI*)

The total capital investment (*TCI*) is obtained by multiplying the purchased equipment cost (*PEC*) by the total installation factor of 2.2 given by Eq. (8) [9].

(8) TCI=2.2PEC

#### 2.3 Estimation of annual cost

Total annual costs (*AC*) [3] include direct annual costs (*DC*) and indirect annual costs (*IC*). In the direct annual costs part, operating labor (operator and supervisor), operating materials (solvent and chemicals), wastewater disposal, maintenance (labor and material) and electricity (fan and pump) are included. In the indirect annual costs part, overhead, administrative charges, property tax, insurance and capital recovery are included.

1) Direct annual cost (*DC*)
  Direct annual cost (*DC*) is defined as expenses related to operating the equipment such as labor and materials.
  Operating labor is estimated at ½-h per 8-h shift. The supervisory labor cost is estimated at 15% of the operating labor cost. Maintenance labor is estimated at ½ -hour per 8-h shift. The operating labor was paid $28.84/hr based on the latest mean hourly wage for plant and system operators; while the maintenance labor was paid $24.48/h based on the latest mean hourly wage for industrial machinery installation, repair, and maintenance workers [10].
  For cost of solvent (water), it should be corresponding to the delivery cost. Here, the value of $0.13/m^3^ ($0.6/1000 gal) is used [11].
  The cost of solvent (*Cs*) is calculated based on Eq. (9). Typically, the fraction of solvent wasted varies from 0.1% to 10% of the total solvent throughput.
  (9) $$Cs=L_{i}WF\left(60\frac{\min}{hr}\right)\left(AOH\right)\left(SUC\right)$$
  Where *WF* is the waste fraction; *SUC* is the solvent unit cost $/gal; *AOH* is annual operating hours, hr; *SUC* is solvent unit cost, $/ft; $L_{i}$ is the water flow rate, gpm;
  The waste water needs to be treated by caustic soda for neutralization before disposal [12]. An intermediate value for the cost of caustic soda of $500/dmt is adopted here [13].
  The cost of chemical replacement (*C*c) is based on the annual consumption of the chemical and can be calculated by Eq. (10).
  (10) Cc=(CUPH)(AOH)(CUC)
  Where *CUC* is the chemical unit cost is in terms of $/lb; *AOH* is the annual operating hours; *CUPH* is chemical used per hour, lbs/hr;
  Caustic soda is used and assumed to be NaOH and the molar ratio between SO_2_ and NaOH is 1:2 [14]. Molar rate of caustic (*MRC*) is calculated by Eq. (11).
  (11) $$MRC=G_{0}P_{SO_{2}}\eta_{SO_{2}}$$
  Here, *G*_0_ is molar rate of flue gas, kmol/s; *P*_SO2_ is the inlet partial pressure of SO_2_; $\eta_{SO_{2}}$ is SO_2_ removal efficiency;
  In terms of the cost for waste water disposal, a value of $2/100 gal is adopted [15], [16].
  Mass flow rate (*MFR*) of salt in the form of Na_2_SO_4_, is calculated as Eq. (12).
  (12) $$MFR_{Na_{2}SO_{4}}=MRC\times M_{Na_{2}SO_{4}}$$
  $MFR_{Na_{2}SO_{4}}$ is the mass flow rate of Na_2_SO_4_, g/s; $M_{Na_{2}SO_{4}}$ is the molecular weight of Na_2_SO_4_, g/mole;
  If the maximum concentration of Na_2_SO_4_ in the wastewater is assumed to be 10% [3], the wastewater volume flow rate (*WVFR*) is calculated as Eq. (13).
  (13) $$WVFR=MFR_{Na_{2}SO_{4}}/0.1/DSS$$
  *WVFR* is wastewater volume flow rate, L/min; *DSS* is the density of Na_2_SO_4_ solution, g/L; Here the density of 10% w/w Na_2_SO_4_ solution at 20 °C is 1090.5 g/L.
  Solvent disposal costs (*C*_ww_) are calculated by Eq. (14).
  (14) $$C_{ww}=L_{i}WF\left(60\frac{\min}{hr}\right)\left(AOH\right)\left(SDC\right)$$
  Where *SDC* is the unit solvent disposal costs, $/gal; $L_{i}WF=WVFR/3.7854$; 3.7854 is the conversion factor from L to gallon.
  Maintenance materials costs are assumed to equal maintenance labor costs.
  Electricity costs include fan to drive the flue gas flow and pump requirements to recirculate the solvent. The electricity cost in industry was $0.675/kWh [17].
  The energy required for the fan ($Energy_{fan}$) can by calculated by Eq. (15).
  (15) $$\;Energy_{fan}=\frac{1.17\times 10^{-4}G_{i}P}{\varepsilon }$$
  Here, $G_{i}$ is the gas flow rate, actual cubic feet per minute (ft^3^/min); P is the total pressure drop through the system, inches of water; ε is the fan motor efficiency chosen as 70%;
  The energy required for the pump ($Energy_{pump}$) is calculated by Eq. (16).
  (16) $$\;Energy_{pump}=\frac{(0.746)\left(2.52\times 10^{-4}\right)L_{i}\left(pressure\right)}{\varepsilon }$$
  Here, 0.746 is the factor used to convert horsepower to kW; pressure is expressed in feet of water;ε is the combined pump motor efficiency chosen as 70%; for the value of pressure, the pump is assumed to work at a pressure of 60 feet of water.
  The cost of electricity (*C*_e_) is then given by Eq. (17).
  (17) $$\;C_{e}=Energy_{fan+pump}\left(AOH\right)\left(COE\right)$$
  Where *COE* is the unit cost of electricity, $/kWh.
2) Indirect annual costs [3]
  Indirect annual cost include overhead, taxes, insurance, general and administrative (G&A), and capital recovery costs.
  Overhead is assumed to be equal to 60% of the sum of operating, supervisory, and maintenance labor, and maintenance materials.
  G&A costs, property tax, and insurance are factored from total capital investment, typically at 2 percent, 1 percent, and 1 percent, respectively.
  Capital recovery cost, *CRC*, is based on an estimated 15 year equipment life. For a 15-year life and an interest rate of 10%, the capital recovery factor is 0.1315. The capital recovery cost is then estimated by Eq. (18).
  (18) CRC=0.1315TCI
3) Total annual cost [3]

Total annual cost (*TAC*) is calculated by adding the direct annual cost and the indirect annual costs through Eq. (19).

(19) TAC=DC+IC
